# Rapid and Visual Detection of SARS-CoV-2 Using Multiplex Reverse Transcription Loop-Mediated Isothermal Amplification Linked With Gold Nanoparticle-Based Lateral Flow Biosensor

**DOI:** 10.3389/fcimb.2021.581239

**Published:** 2021-07-14

**Authors:** Xu Chen, Qingxue Zhou, Shijun Li, Hao Yan, Bingcheng Chang, Yuexia Wang, Shilei Dong

**Affiliations:** ^1^ The Second Clinical College, Guizhou University of Traditional Chinese Medicine, Guiyang, China; ^2^ Central Laboratory of the Second Affiliated Hospital, Guizhou University of Traditional Chinese Medicine, Guiyang, China; ^3^ Laboratory of Bacterial Infectious Disease of Experimental Centre, Guizhou Provincial Centre for Disease Control and Prevention, Guiyang, China; ^4^ Clinical Laboratory, Hangzhou Women’s Hospital, Hangzhou, China; ^5^ Department of Microbiology, Zhejiang Provincial Center for Disease Control and Prevention, Hangzhou, China; ^6^ TCM Research Institute, Zhejiang Chinese Medical University, Hangzhou, China; ^7^ Department of Clinical Laboratory, Zhejiang Hospital, Hangzhou, China

**Keywords:** lateral flow biosensor, reverse transcription-loop-mediated isothermal amplification, limit of detection, COVID-19, SARS-CoV-2

## Abstract

**Background:**

Severe acute respiratory syndrome coronavirus 2 (SARS-CoV-2) is a novel coronavirus that has caused the outbreak of coronavirus disease 2019 (COVID-19) all over the world. In the absence of appropriate antiviral drugs or vaccines, developing a simple, rapid, and reliable assay for SARS-CoV-2 is necessary for the prevention and control of the COVID-19 transmission.

**Methods:**

A novel molecular diagnosis technique, named multiplex reverse transcription loop-mediated isothermal amplification, that has been linked to a nanoparticle-based lateral flow biosensor (mRT-LAMP-LFB) was applied to detect SARS-CoV-2 based on the SARS-CoV-2 *RdRp* and *N* genes, and the mRT-LAMP products were analyzed using nanoparticle-based lateral flow biosensor. The mRT-LAMP-LFB amplification conditions, including the target RNA concentration, amplification temperature, and time were optimized. The sensitivity and specificity of the mRT-LAMP-LFB method were tested in the current study, and the mRT-LAMP-LFB assay was applied to detect the SARS-CoV-2 virus from clinical samples and artificial sputum samples.

**Results:**

The SARS-CoV-2 specific primers based on the *RdRp* and *N* genes were valid for the establishment of mRT-LAMP-LFB assay to detect the SARS-CoV-2 virus. The multiple-RT-LAMP amplification condition was optimized at 63°C for 30 min. The full process, including reaction preparation, viral RNA extraction, RT-LAMP, and product identification, could be achieved in 80 min. The limit of detection (LoD) of the mRT-LAMP-LFB technology was 20 copies per reaction. The specificity of mRT-LAMP-LFB detection was 100%, and no cross-reactions to other respiratory pathogens were observed.

**Conclusion:**

The mRT-LAMP-LFB technique developed in the current study is a simple, rapid, and reliable method with great specificity and sensitivity when it comes to identifying SARS-CoV-2 virus for prevention and control of the COVID-19 disease, especially in resource-constrained regions of the world.

## Introduction

Severe acute respiratory syndrome coronavirus 2 (SARS-CoV-2), a non-segmented positive-sense RNA genome virus, is a novel coronavirus that causes the outbreak of respiratory disease (COVID-19) all over the world (Bao et al., 2020; Zhang, 2020). In the 21st century, two important coronaviruses, severe acute respiratory syndrome coronavirus (SARS-CoV) and Middle East respiratory syndrome coronavirus (MERS-CoV), have severely threatened public health (in 2003 and 2012, respectively) (Chen, 2020; Wang et al., 2020). Since December 2019, the novel SARS-CoV-2 coronavirus has been found in many countries around the world and was declared as a disease of “public health emergency of international concern” by the World Health Organization (WHO) (Rothe et al., 2020). Most patients infected with SARS-CoV-2, present with acute onset of fever, cough, dyspnea, and radiological evidence of ground-glass lung opacities compatible with atypical pneumonia (Tu et al., 2020). Not only that, asymptomatic or mildly symptomatic cases have also been reported (Coronaviridae Study Group of the International Committee on Taxonomy of Viruses, 2020; Jiang et al., 2020). Owning to the current disease situation, the SARS-CoV-2 virus has been becoming the third coronavirus posing significant threats to public health worldwide. In the absence of appropriate antiviral drugs or vaccines, developing a reliable, simple, and rapid assay for SARS-CoV-2 is necessary for the prevention and control of the COVID-19 transmission.

The size of SARS-CoV-2 genome is ~30 kilobases and encodes ~9860 amino acids, which has been classified as a beta coronavirus (Ji, 2020; Younes et al., 2020). The genome of SARS-CoV-2 is arranged in the order of 5’-untranslated region (UTR), replicase complex (*ORF1a/b*), spike gene (*S* gene), *E* gene, *M* gene, *N* gene, 3’ UTR, and several unidentified non-structural open reading frames (van Kasteren et al., 2020; Younes et al., 2020). Since the outbreak of COVID-19, real-time reverse transcription-polymerase chain reaction (RT-PCR) is the most robust and widely used technology for the detection of SARS-CoV-2 in hospitals and other medical institutions (Corman et al., 2020; Tahamtan and Ardebili, 2020; Zhen et al., 2020). However, RT-PCR assays require special experimental instruments, are time-consuming, and require skilled personnel, which may not be readily available in many resource-poor settings. Therefore, a cost-effective, simple, reliable, rapid, sensitive, and specific assay for the identification of SARS-CoV-2 is urgently developed to improve the detection capability and prevent the spread of COVID-19.

To overcome the drawbacks of RT-PCR detection, a wide variety of isothermal amplification-based methods have been developed for use in molecular identification (Wang et al., 2015; Wang et al., 2017). Loop-mediated isothermal amplification (LAMP), as a reliable, sensitive, and rapid assay with low equipment cost, has been widely applied to detect many pathogens, including SARS-CoV, MERS-CoV, and influenza virus (Huang et al., 2018; Kim et al., 2019; Ravina et al., 2020). LAMP products have been analyzed by various methods, including visual inspection of color changes, turbidimetry changes, and fluorescence dye (Notomi et al., 2000; Wang et al., 2019; Lu et al., 2020). However, these detection techniques require special apparatus and reagents. To overcome this defect, a target-specific, visual and simple nanoparticle-based lateral flow biosensor (LFB) detection method was successfully designed and applied to analyze mRT-LAMP products (Jiao et al., 2019; Li et al., 2019; Wang et al., 2019). In this study, a multiplex reverse transcription LAMP technique linked to an LFB detector (mRT-LAMP-LFB) was developed for the simple, specific, reliable, sensitive, and visual identification of SARS-CoV-2 by targeting the RNA-dependent RNA polymerase gene (*RdRp* gene) and nucleocapsid protein gene (*N* gene) (Chen et al., 2020; Huang et al., 2020). The optimal amplification conditions and feasibility of the mRT-LAMP-LFB assay were confirmed with SARS-CoV-2 pseudo-virus, clinical samples, and artificial sputum samples.

## Materials and Methods

### Materials Instruments

Viral RNA extraction kits (QIAamp Viral RNA minikits; Qiagen, Hilden, Germany) (Cat NO. 52906) were purchased from Qiagen (Beijing, China). Universal isothermal amplification kits, AMV Reverse Transcriptase, colorimetric indicator (malachite green, MG), and biotin-14-dCTP were obtained from Bei-Jing HaiTaiZhengYuan. Co., Ltd. (Beijing, China). The LFB materials, including the backing card, sample pad, absorbent pad, conjugate pad, and nitrocellulose membrane (NC), were purchased from Jie-Yi Biotechnology. Co., Ltd. (Shanghai, China). Anti-FAM (rabbit anti-fluorescein antibody) and biotin-BSA (biotinylated bovine serum albumin) were purchased from Abcam. Co., Ltd. (Shanghai, China). Dye (Crimson red) streptavidin-coated polymer nanoparticles (129 nm, 10 mg ml^-1^; 100 mM borate, pH 8.5, with 0.1% BSA, 0.05% Tween 20 and 10 mM EDTA) were purchased from Bangs Laboratories, Inc. (Indiana, USA).

### Design of RT-LAMP Primers

**Figure 1:**
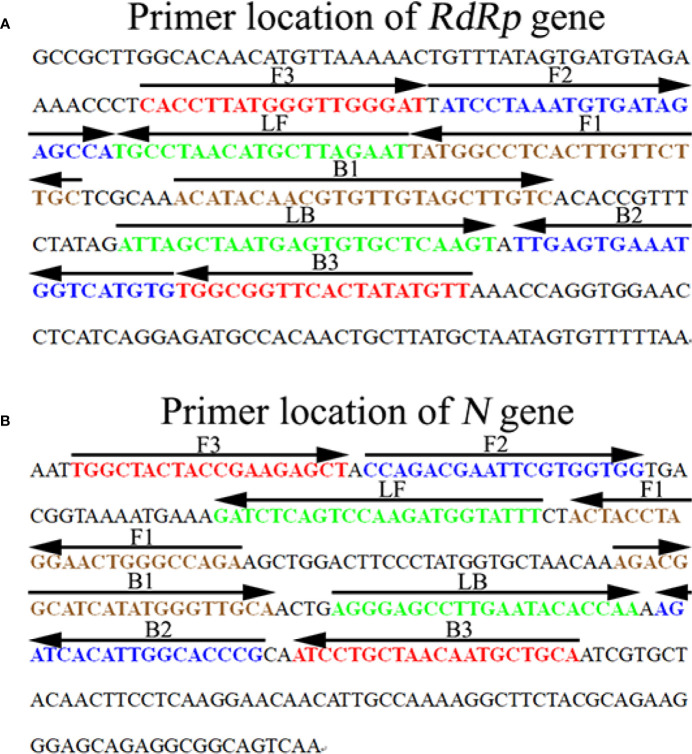
Sequence and location of the *RdRp* **(A)** and *N* **(B)** genes used to design SARS-CoV-2 mRT-LAMP primers. The nucleotide sequence of the sense strand of the *RdRp* and *N* is shown in the diagram. Right arrows and left arrows indicate sense and complementary sequences which were used in the current study, respectively.

Based on the reaction mechanism of LAMP, two sets of specific primers were designed according to the target genes *RdRp* and *N* (GenBank Accession No. NC_045512.2), respectively. The primers were designed with Primer Explorer V5 (http://primerexplorer.jp/e/; Eiken Chemical Co., Ltd., Tokyo, Japan) online primer design software and checked with the basic local alignment search tool (BLAST). The primer positions are shown in **Figure 1**, and the *RdRp* and *N* genes sequence alignment among seven human coronaviruses (SARS-CoV-2, SARS-CoV, MERS-CoV, HCoV-HKU-1, HCoV-NL63, HCoV-OC43, and HCoV-229E) are shown in **Supplementary Figure 1**. The primer sequences and modifications are shown in **Table 1**. All of the primers were synthesized by TsingKe Biotech Co., Ltd. (Beijing, China) with HPLC purification grade.

**Table 1:** The primers used in the present study.

Primer name	Sequence and modifications	Length	Gene
F3	5′-CACCTTATGGGTTGGGAT-3′	18 nt	*RdRp*
B3	5′-AACATATAGTGAACCGCCA-3′	19 nt	
FIP	5′-GCAAGAACAAGTGAGGCCATA-ATCCTAAATGTGATAGAGCCA-3′	42 mer	
BIP FIP*	5′-ACATACAACGTGTTGTAGCTTGTC-CACATGACCATTTCACTCAA-3′ 5′-FAM-GCAAGAACAAGTGAGGCCATA-ATCCTAAATGTGATAGAGCCA-3′	44 nt 42 mer	
LF	5′-ATTCTAAGCATGTTAGGCA-3′	19 nt	
LB LF*	5′-ATTAGCTAATGAGTGTGCTCAAGT-3′ 5′-Biotin-ATTCTAAGCATGTTAGGCA-3′	24 nt 19 nt	
F3	5′-TGGCTACTACCGAAGAGCT-3′	19 nt	*N*
B3	5′-TGCAGCATTGTTAGCAGGAT-3′	20 nt	
FIP	5′-TCTGGCCCAGTTCCTAGGTAGT-CCAGACGAATTCGTGGTGG-3′	41 nt	
BIP	5′-AGACGGCATCATATGGGTTGCA-CGGGTGCCAATGTGATCT-3′	40 nt	
FIP*	5′-Dig-TCTGGCCCAGTTCCTAGGTAGT-CCAGACGAATTCGTGGTGG-3′	41 nt	
LF	5′-AAATACCATCTTGGACTGAGATC-3′	23 nt	
LB	5′-AGGGAGCCTTGAATACACCAA-3′	21 nt 23 nt	
LF*	5′-Biotin-AAATACCATCTTGGACTGAGATC-3′		

RdRp-FIP*, 5′-labeled with FAM when used in LAMP-LFB assay; RdRp-LF*, 5′-labeled with biotin when used in LAMP-LFB assay;

N-FIP*, 5′-labeled with Dig when used in the LAMP-LFB assay; N-LF*, 5′-labeled with biotin when used in the LAMP-LFB assay.

FAM, 6-carboxy-fluorescein; Dig, digoxigenin; nt, nucleotide; mer, monomeric unit.

### SARS-CoV-2 RNA Standard and Artificial SARS-CoV-2 Virus Preparation

The SARS-CoV-2 RNA standard material was obtained from the Chinese Academy of Metrology (Code NO. GBW (E) 091089). The RNA transcripts contained *ORF1ab* gene segment (13201-15600), complete *E* gene, and *N* gene (GenBank NO. NC_045512), and the concentration of RNA was measured by absolute quantitative digital PCR.

The pseudo-virus for the positive quality control agent was obtained from TsingKe Biotech Co., Ltd. (Beijing, China) (Cat NO. TSV2614), which was made with 293T cell cultures and included segments of the ORF1a/b gene (genome coordinates: 13237-13737, 15231-15729), M Gene (genome coordinates: 26523-27191), E Gene (genome coordinates: 26245-26472), and N Gene (genome coordinates: 28274-29533). The pseudo-virus of SARS-CoV and MERS-CoV were obtained from TsingKe Biotech Co., Ltd. (Cat NO. TSV2589; Cat NO.TSV2575).

### RNA Template Preparation

In the current study, the viral RNA comes from both pseudo-virus (TsingKe Biotech Co., Ltd) and clinical samples were obtained using Viral RNA Extraction Kits (Qiagen, Hilden, Germany) in accordance with the manufacturer’s instructions. The RNA templates were stored at -80°C before use. The concentration was assayed using quantitative PCR with RNA standard. Then, 10-fold serial dilutions of the pseudo-viruses ranging from 1×10^4^copies/μl to 1 copy/μl were prepared.

### Gold Nanoparticle-Based Lateral Flow Biosensor Preparation

The LFB platform was prepared according to a previous report (Cheng et al., 2019). Briefly, the LFB contained four components: an absorbent pad, NC membrane, sample pad, and conjugate pad (Jie-Yi Biotechnology. Co., Ltd.). The components were assembled orderly on a backing card. The capture reagents, including anti-FAM, anti-Dig, and biotin-BSA (Abcam. Co., Ltd.), were immobilized by physical adsorption on the reaction regions. Then, anti-FAM was immobilized at test line 1 (TL1) (*RdRp*), and anti-Dig was immobilized at test line 2 (TL2) (*N*), while biotin-BSA was immobilized at the control line (CL); each line was separated by 5 mm. SA-PNPs (dye streptavidin-coated polymer nanoparticles) were gathered on the conjugate pad. The prepared biosensors were preserved in a plastic box with a desiccant gel at room temperature before use.

### The Standard RT-LAMP Reaction

The single RT-LAMP reactions for *RdRp* or *N* were performed in 25 μl reaction systems as previously described. Briefly, 0.4 μM of each outer primer (F3 and B3), 0.8 μM of each loop primer (LF* and LB), 1.6 μM of each inner primer (FIP* and BIP), 0.4 mM of biotin-14-dCTP, 1 μl (8 U) of *Bst* DNA polymerase (New England Biolabs, USA), 1 μl (10 U) of AMV Reverse Transcriptase (New England Biolabs, USA), 12.5 μl of 2 × reaction buffer [40 mM Tris-HCl (pH 8.8), 40 mM of KCl, 16 mM of MgSO_4_, 20 mM of (NH_4_)_2_SO_4_, 2 M of betaine, and 0.2% Tween-20] (HuiDeXin Bio-technique, Tianjin, China), and 1 × 10^4^ copies of the RNA template were added to a tube. The mixtures were incubated at 63°C for 1 h. Viral RNA from SARS-CoV (pseudo-virus), MERS-CoV (pseudo-virus), and double distilled water (DW) were used as negative controls (NCs). The mRT-LAMP reaction was performed in a one-step reaction in a 25 μl reaction system containing 12.5 μl of 2 × reaction buffer; 0.2 μM each outer primer, *RdRp*-F3, *RdRp*-B3, *N*-F3, and *N*-B3; 0.4 μM each loop primer, *RdRp*-LF*, *RdRp*-LB, *N*-LF* and *N*-LB; 0.8 μM each inner primer, *RdRp*-FIP*, *RdRp*-BIP, *N*-FIP* and *N*-BIP; 0.4 mM biotin-14-dCTP; 1 μl (8 U) of *Bst* DNA polymerase (New England Biolabs, USA); 1 μl (8 U) of AMV Reverse Transcriptase (New England Biolabs, USA); and 1 × 10^4^ copies of RNA template. The reaction conditions were carried out as described above.

### RT-LAMP Products Detection

The monitoring techniques, including 2% agarose gel electrophoresis, visual detection reagents MG (VDR, Haitai-Zhengyuan biotech, Co. Ltd. Beijing, China), and lateral flow biosensor (LFB) methods, were applied for the determination and verification of the *RdRp*-RT-LAMP, *N*-RT-LAMP, and mRT-LAMP products. For the products amplified effectively, the agarose gel presented ladder-like bands, and the color changed from colorless to light green in the MG assay. However, there have no bands in gel electrophoresis, and the color remains colorless in negative and blank controls. The strategy of visualization of RT-LAMP products with LFB was as previously described (Gong et al., 2019).

### Temperature Optimization of the RT-LAMP Assays

To confirm the optimal amplification temperature for *RdRp*-RT-LAMP and *N*-RT-LAMP, the pseudo-virus of SARS-CoV-2-*RdRp*-*N* was used as a positive control at a concentration of 1×10^4^ copies per reaction, and the RT-LAMP amplifications were monitored by a real-time turbidity technique. Reaction temperatures ranging from 60 to 67°C with 1°C intervals were tested. The curves of DNA concentrations of each amplified product were exhibited in the graph. Turbidity > 0.1 was considered as positive. Three replicates were tested for each temperature.

### Optimization of the Amplification Time for the mRT-LAMP-LFB Assay

To optimize the reaction time of mRT-LAMP-LFB, four amplification times (20, 30, 40, and 50 min) were evaluated. The mRT-LAMP-LFB reactions were carried out as described above, and the results were tested by LFB. Each reaction time was tested at least three times.

### Analytical Sensitivity of mRT-LAMP-LFB Assays

The sensitivity of each RT-LAMP-LFB reaction (*RdRp*-RT-LAMP-LFB, *N*-RT-LAMP-LFB, and mRT-LAMP-LFB) was determined using pseudo-virus of SARS-CoV-2 with ten-fold serial dilutions range from 1×10^4^ copies to 1 copy. The RT-LAMP reactions were carried out as described above, and the results were tested using visual detection reagents (MG) and LFB. The limit of detection (LoD) of single and multiplex reactions was verified as the last dilution of each positive test. The LoD of RT-PCR technology using Applied Biosystems™ 7500 Real-Time PCR System (Life Technologies, Singapore) with Novel Coronavirus Nucleic Acid Diagnostic Real-Time RT-PCR Kit (Sansure biotech Inc, China) was also tested in the current study. Three replicates were tested for each dilution.

### Specificity Analysis of mRT-LAMP-LFB Detection

To evaluate the specificity of the mRT-LAMP-LFB assay, pseudo-viruses of SARS-CoV-2, SARS-CoV-2 positive clinical samples, and other pathogens (**Table 2**) were used for mRT-LAMP detection, and all of the results were tested using the LFB method. All examinations were confirmed at least three times.

**Table 2:** Pathogens used in the current study.

No.	Pathogen species	Pathogen name	Source of pathogens^a^	No. of strains	RT-LAMP-LFB result^b^	
					*RdRp*	*N*
	**Coronavirus**					
1	SARS-CoV-2 (pseudo-virus)	2019-nCoV-ab II EMN	TsingKe Biotech Co., Ltd. (Beijing, China)	1	P	P
2	SARS-CoV-2 (nucleic acid samples)	ZJCDC-2019-nCoV-52; -85;-86;-90-120;-123;-134;-152;-189;-190; ZJ-2019-nCoV-304;-305	ZJCDC and 1^st^ ZJUSM	12	P	P
3	SARS-CoV (pseudo-virus)	SARS-ORF1a-N	TsingKe Biotech Co., Ltd. (Beijing, China)	1	N	N
4	MERS-CoV (pseudo-virus)	MERS-abEN	TsingKe Biotech Co., Ltd. (Beijing, China)	1	N	N
5	Human coronavirus HKU1	Quality control sample	DAAN Gene Co., Ltd. (Guangzhou, China)	1	N	N
6	Human coronavirus HCoV-NL63	Quality control sample	DAAN Gene Co., Ltd. (Guangzhou, China)	1	N	N
7	Human coronavirus OC43	Quality control sample	DAAN Gene Co., Ltd. (Guangzhou, China)	1	N	N
8	Human coronavirus 229E	Quality control sample	DAAN Gene Co., Ltd. (Guangzhou, China)	1	N	N
	**Other pathogens**					
9	H1N1	ZJH-H1N1-57	Zhejiang Hospital	1	N	N
10	H3N2 (nucleic acid sample)	GZCDC-H3N2-14	GZCDC	1	N	N
11	H5N1 (nucleic acid sample)	GZCDC-11-H5N1	GZCDC	1	N	N
12	H7N9 (nucleic acid sample)	GZCDC-5-H7N9	GZCDC	1	N	N
13	Influenza B	ZJH Influenza B-115	Zhejiang Hospital	1	N	N
14	Respiratory syncytial virus type A	Quality control sample	DAAN Gene Co., Ltd. (Guangzhou, China)	1	N	N
15	Respiratory syncytial virus type B	Quality control sample	DAAN Gene Co., Ltd. (Guangzhou, China)	1	N	N
16	Human rhinovirus	Quality control sample	DAAN Gene Co., Ltd. (Guangzhou, China)	1	N	N
17	Adenoviruses	Quality control sample	DAAN Gene Co., Ltd. (Guangzhou, China)	1	N	N
18	*Mycoplasma pneumoniae*	ZJH-MP-594	Zhejiang Hospital	1	N	N
19	*Mycobacterium tuberculosis*	GZCDC-MTB-564	GZCDC	1	N	N
20	*Pseudomonas aeruginosa*	ATCC 27853	ATCC	1	N	N
21	*Klebsiella pneumonia*	ZJH-KP-104	Zhejiang Hospital	1	N	N
22	*Streptococcus pneumoniae*	ZJH-SP-016	Zhejiang Hospital	1	N	N
23	*Mycoplasma pneumonia* M129/FH	2^nd^ GZUTCM-MP-102	2^nd^ GZUTCM	1	N	N
24	*Haemophilus influenza*	ATCC49247	ATCC	1	N	N
25	*Streptococcus pyogenes*	ZJH-SP-1087	Zhejiang Hospital	1	N	N
26	*Acinetobacter baumannii*	ZJH-AB-984	Zhejiang Hospital	1	N	N
27	*Staphylococcus aureus*	ZJH-SA-065	Zhejiang Hospital	1	N	N
28	*Cryptococcus neoformans*	ATCC14053	ATCC	1	N	N
29	*Candida glabrata*	ZJH-CG-057	Zhejiang Hospital	1	N	N
30	*Hemophililus parainfluenza*	GZCDC-HP-045	GZCDC	1	N	N
31	*Shigella boydii*	GZCDC-SB-107	GZCDC	1	N	N
32	Enteropathogenic *Escherichia coli*	GZCDC-EPEC-045	GZCDC	1	N	N
33	*Bordetella pertussis*	GZCDZ-BP-052	GZCDC	1	N	N
34	*Bordetella parapertussis*	GZCDC-BP-0094	GZCDC	1	N	N
35	*Bacillus cereus*	2^nd^ GZUTCM-BC-037	2^nd^ GZUTCM	1	N	N
36	*Listeria monocytogenes*	2^nd^ GZUTCM-LM-025	2^nd^ GZUTCM	1	N	N
37	*Shigella flexneri*	2^nd^ GZUTCM-SF-018	2^nd^ GZUTCM	1	N	N
38	*Leptospira interrogans*	GZCDC-LI-005	GZCDC	1	N	N

a ZJCDC, Zhejiang Provincial Center for Disease Control and Prevention; 1^st^ ZJUSM, The First Affiliated Hospital, Zhejiang University School of Medicine; ZJCCL, Zhejiang Center for Clinical Laboratories; 2^nd^ GZUTCM, The Second Affiliated Hospital, Guizhou University of Traditional Chinese Medicine; GZCDC, Guizhou Provincial Center for Disease Control and Prevention; ATCC, American Type Culture Collection.

b P, Positive; N, Negative.

### Application of the mRT-LAMP-LFB Method to Analyze the Clinical Samples and Artificial Sputum Samples

To verify the applicability of the mRT-LAMP-LFB assay for detecting SARS-CoV-2, one hundred and ten clinical nasopharyngeal swab specimens were collected from suspected SARS-CoV-2 infected patients, and sixty artificial sputum samples (randomly added 100 copies of SARS-CoV-2 pseudo-viruses in each 200 μl artificial sputum sample) were used in the current study. The artificial sputum samples were pretreated with N-acetyl-L-cysteine-2% NaOH. The initial process of all specimens was handled in a validated biological safety cabinet, and performed by staff trained with appropriate personal protective equipment. The clinical samples and artificial sputum samples were detected for SARS-CoV-2 using RT-PCR and mRT-LAMP-LFB methods. The mRT-LAMP detection was as described above. The Novel Coronavirus Nucleic Acid Diagnostic Real-Time RT-PCR Kit (Sansure biotech Inc, China) was used as the reference standard, which was recommended by the Chinese Center for Disease Control and Prevention. The RT-PCR detection was performed with Applied Biosystems™ 7500 Real-Time PCR System (Life Technologies, Singapore). A threshold cycle (Ct value) < 38 was determined to indicate a positive result. The mRT-LAMP-LFB and RT-PCR assays were performed simultaneously in a biosafety level 2 laboratory, as detailed in the WHO Laboratory biosafety manual, third edition. The mRT-LAMP-LFB detection was performed as described above.

## Results

COVID-19 is a newly emerging, life-threatening respiratory disease caused by a novel coronavirus SARS-CoV-2, and it has had a significant impact on public health and the economy worldwide (Bao et al., 2020; She et al., 2020). The purpose of the current study is to develop a reliable, rapid, sensitive, and easy-to-use assay for SARS-CoV-2.

### Verification and Analysis of RT*-*LAMP Products

**Figure 2:**
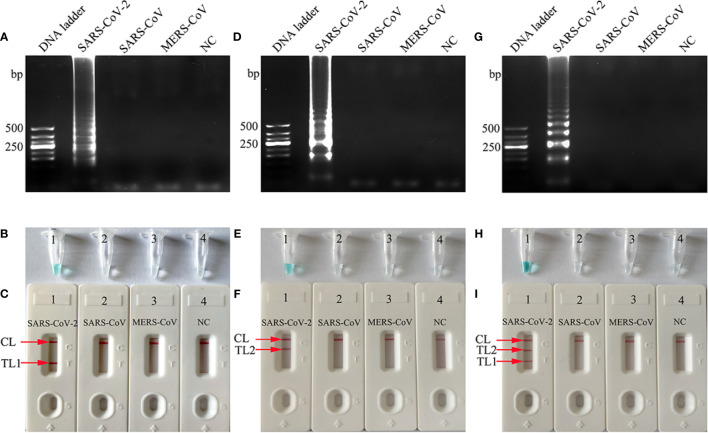
Determination and verification of mRT-LAMP products. The *RdRp*-, *N*-, or mRT-LAMP mixtures, containing 1 × 10^4^ copies of the RNA template, were incubated at a constant temperature of 65°C for 1 h, and the RT-LAMP products were identified with 2% agarose gel electrophoresis **(A, D, G)**, visual detection reagents **(B, E, H)** and lateral flow biosensor **(C, F, I)**. Viral RNA from pseudo-virus SARS-CoV, pseudo-virus MERS-CoV, and double distilled water (DW) were used as negative controls (NCs). Lane DNA ladder: 500 bp DNA ladder, the ladder-like bands indicate positive RT-LAMP amplification, the color changed from colorlessness to bright green indicates positive nucleic acid amplification. CL and TL1 appeared crimson red bands, indicating positive results of *RdRp*-RT-LAMP products, CL and TL2 presented crimson red bands, indicating positive results of *N*-RT-LAMP products, three crimson red bands (CL, TL1, and TL2) appeared indicating positive results of mRT-LAMP amplification.

To confirm the amplification with the two sets of LAMP primers, the *RdRp*-, *N*-, or mRT-LAMP mixtures were incubated at a constant temperature of 65°C for 1 h. Then, the *RdRp*-, *N*-, and mRT-LAMP products were analyzed with 2% agarose gel electrophoresis, colorimetric indicator (MG), and lateral flow biosensor (LFB), respectively. The ladder-liker bands of agarose gel were observed in the positive amplification, but not in the negative controls (**Figures 2A, D, G**). The color of the positive results in the *RdRp*-, *N*-, and mRT-LAMP reactions changed from colorlessness to bright green, while the negative reactions remained colorless (**Figures 2B, E, H**). LFB was used for further confirmation of *RdRp*-, *N*-, and mRT-LAMP. For *RdRp*-RT-LAMP detection, two crimson red bands (CL and TL1) appeared, indicating positive results, CL and TL2 were visible for *N*-RT-LAMP, indicating successful amplification, while the negative controls only appeared as a crimson red line (CL) in the biosensor (**Figures 2C, F, I**). Therefore, the results suggested that the two sets of RT-LAMP primers for *RdRp* and *N* detection were valid for the development of the mRT-LAMP assay.

### Optimal Reaction Temperature for *RdRp*-RT-LAMP and *N*-RT-LAMP Amplification

**Figure 3:**
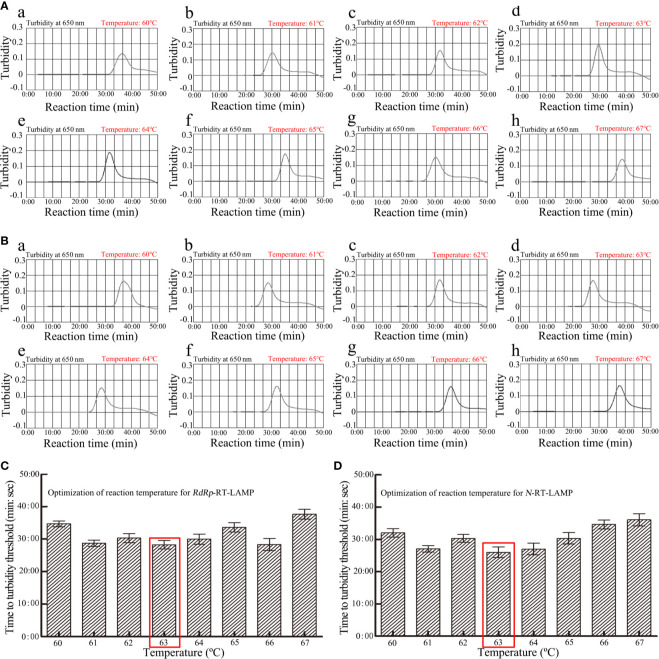
Optimization of amplification temperature for *RdRp* -LAMP **(A)** and *N*-LAMP **(B)** primer sets. The LAMP amplifications for detection of *RdRp* **(A)** and *N* **(B)** were monitored through real-time turbidity and the corresponding curves of amplicons were displayed in the graphs. The threshold value was 0.1 and the turbidity>0.1 was considered as positive. 8 kinetic graphs were obtained at different temperatures (60-67°C, 1°C intervals) with 1×10^4^ copies target genomic RNA per reaction. **(C)** Optimization of reaction temperature for *RdRp*-RT-LAMP; **(D)** Optimization of reaction temperature for *N*-RT-LAMP.

The reaction temperature is crucial for RT-LAMP amplification. In this study, the reaction temperature of *RdRp*- and *N*-LAMP amplification was tested at different temperatures (60 to 67°C with 1°C intervals) with genomic templates (1×10^4^ copies) from the pseudo-virus of SARS-CoV-2. The RT-LAMP amplification protocol was as described above, the *RdRp*- and *N*-LAMP amplification were monitored by means of real-time turbidity technique, and the kinetics graphs were recorded from all temperatures. The results showed that the faster amplifications of *RdRp*-RT-LAMP were obtained for detection temperature range from 63 to 64°C, and 62 to 63°C for the *N*-RT-LAMP reactions (**Figure 3**). Hence, the amplification temperature of 63°C was considered as optimal temperature for the rest of multiple-RT-LAMP reactions in the current study.

### Optimization of Amplification Time for mRT-LAMP-LFB Assay

**Figure 4:**
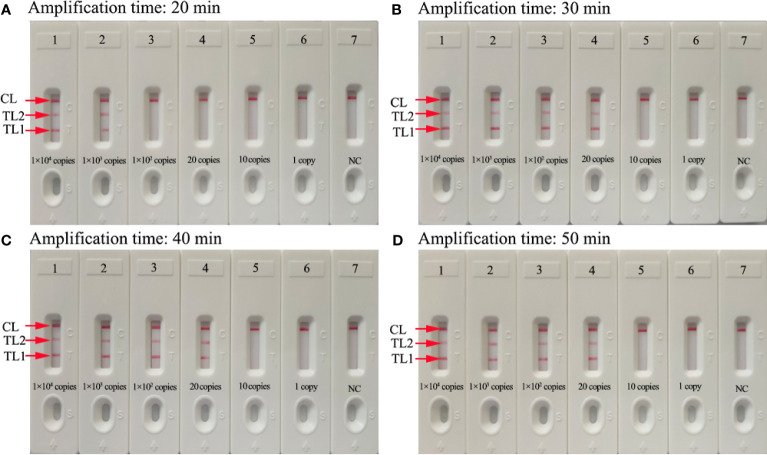
Optimization of the amplification time for mRT-LAMP-LFB detection. Different amplification times (**A**, 20 min, **B**, 30 min, **C**, 40 min, **D**, 50 min) were tested at 63°C. Biosensors 1-7 represent SARS-CoV-2 genomic RNA levels of 1×10^4^ copies, 1×10^3^ copies, 1×10^2^ copies, 20 copies, 10 copies, and 1 copy per reaction and blank control (DW), respectively. The best sensitivity was observed when the amplification lasted for 30 min **(B)**.

To obtain an optimal reaction time for mRT-LAMP, four amplification times (20, 30, 40, and 50 min) were tested at the 63°C amplification temperature. The results showed that the LoD of the genomic RNA templates (20 copies) was detected when the mRT-LAMP amplification lasted 30 min (**Figure 4**). Hence, a reaction time of 30 min was considered the optimal amplification time for mRT-LAMP detection. In summary, the whole detection procedure, including reaction preparation (approximately 10 min), target genomic RNA preparation (30 min), mRT-LAMP (30 min), and analysis of results (approximately 2 min), could be completed within 80 min.

### Sensitivity of *RdRp*-, *N*-, and mRT-LAMP Detection

**Figure 5:**
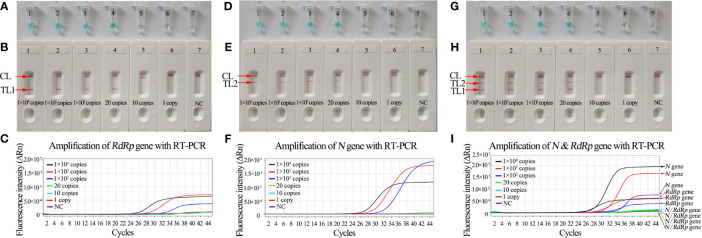
Sensitivity analysis of *RdRp*-, *N*-, and mRT-LAMP detection with serial dilutions of RNA extracted from pseudo-virus SARS-CoV-2. The LoD of RT-LAMP for detecting SARS-CoV-2 was analyzed with visual detection reagents (MG) and lateral flow biosensors. **(A, B)** Sensitivity analysis of *RdRp*-RT-LAMP reaction. Tubes A1-A7 (Biosensors B1-B7) represent the genomic RNA amounts of 1×10^4^ copies, 1×10^3^ copies, 1×10^2^ copies, 20 copies, 10 copies, and 1 copy per reaction and blank control (DW), respectively. The LoD of *RdRp*-RT-LAMP detection was 20 copies of RNA template per reaction. **(C)** Sensitive of *RdRp*-RT-PCR detection (1×10^4^ copies to 1 copy). The LoD of *RdRp*-RT-PCR detection was 100 copies of RNA template per reaction. **(D, E)** Sensitivity analysis of *N*-RT-LAMP reaction. Tubes D1-D7 (Biosensors E1-E7) represent the genomic RNA amounts of 1×10^4^ copies, 1×10^3^ copies, 1×10^2^ copies, 20 copies, 10 copies, and 1 copy per reaction and blank control (DW), respectively. The LoD of *N*-RT-LAMP detection was 20 copies of RNA template per reaction. **(F)** Sensitive of *N*-RT-PCR detection (1×10^4^ copies -1 copy). The LoD of *N*-RT-PCR detection was 100 copies of RNA template per reaction. **(G, H)** Tubes G1-G7 (Biosensors H1-H7) represent the genomic RNA amounts of 1×10^4^ copies, 1×10^3^ copies, 1×10^2^ copies, 20 copies, 10 copies, and 1 copy per reaction and blank control (DW), respectively. The LoD of the mRT-LAMP assay for *RdRp* and *N* detection was 20 copies of RNA template per reaction. **(I)** Sensitive of mRT-PCR detection (1×10^4^ copies to 1 copy). The LoD of mRT-PCR detection was 100 copies of RNA template per reaction.

The sensitivity of *RdRp*-, *N*-, and mRT-LAMP detection was evaluated with serially diluted pseudo-virus RNA range from 1×10^4^ copies to 1 copy. The RT-LAMP amplification products were analyzed by visual inspection with MG reagents and lateral flow biosensors. The CL and TL1 lines appeared on the biosensor, showing positive results for the *RdRp*-RT-LAMP assay, and two crimson lines (CL and TL2) were observed on the biosensor, indicating positive results for *N-*RT-LAMP detection. The CL, TL1, and TL2 bands simultaneously became crimson on the biosensor, reporting positive results for the *RdRp* and *N* genes. For the negative controls, only the CL line appeared on the biosensors. The results showed that the LoD of mRT-LAMP was 20 copies per reaction, which was the same as the LoD of the *RdRp*- and *N*-RT-LAMP assay (**Figures 5A, B, D, E, G, H**). Meanwhile, the sensitivity of RT-PCR technique was also tested in the current study, the results indicated that the LoD of RT-PCR was 100 copies per reaction (**Figures 5C, F, I**).

### Specificity of the mRT-LAMP Assay

The specificity of mRT-LAMP detection was confirmed with pseudo-viruses of SARS-CoV-2, 12 clinical SARS-CoV-2-positive samples, and 36 other pathogens (**Table 2**). The process of mRT-LAMP amplification, as described above. The genomic RNA extracted from SARS-CoV-2 presented positive results. Other pathogens and the blank control showed negative results (**Table 2**). Hence, the results confirmed that the mRT-LAMP-LFB method could accurately identify SARS-CoV-2 from other pathogens.

### Feasibility of the mRT-LAMP-LFB Method Using Clinical Samples

To further demonstrate the feasibility of mRT-LAMP-LFB as a valuable method for the detection of SARS-CoV-2, 110 clinical nasopharyngeal swab specimens and 60 artificial sputum samples (randomly added 100 copies of SARS-CoV-2 pseudo-viruses in each 200 μl artificial sputum sample) were simultaneously tested by mRT-LAMP-LFB and RT-PCR. Among them, 12 clinical samples and 35 artificial sputum samples had been confirmed as SARS-CoV-2 through RT-PCR and mRT-LAMP-LFB, respectively (**Table 3**). The Cq values of RT-PCR and mRT-LAMP-LFB detection results were shown in **Supplementary Table 1**. These results suggested that the mRT-LAMP-LFB assay established in the current study could be used as an advanced tool to detect SARS-CoV-2.

**Table 3:** Comparison of RT-PCR and mRT-LAMP-LFB methods to identify SARS-CoV-2 in clinical samples and artificial sputum samples.

Detection method	Clinical samples (n = 110)			Artificial sputum samples (n = 60)		
	Positive	Negative	Time consumption	Positive	Negative	Time consumption
RT-PCR	12 (Ct<38)	98	~150 min	35 (Ct<38)	25	~150 min
mRT-LAMP-LFB	12	98	Within 80 min	35	25	Within 80 min

## Discussion

SARS-CoV-2 is the seventh coronavirus that causes human infections. Like SARS-CoV and MERS-CoV, this virus has the ability to cause lethal pneumonia (Chiappelli, 2020). Moreover, it has a stronger human-to-human transmission capacity than the above two coronaviruses (Ki, 2020; Wilson and Chen, 2020). Until now, up to 140 million COVID-19 cases have been confirmed, including more than 3 million deaths (www.who.int/emergencies/diseases/novel-coronavirus-2019).

The main findings of the current study are that we established a simple, sensitive, reliable, and rapid assay with great specificity and low equipment cost for SARS-CoV-2 by mRT-LAMP-LFB. To avoid false-positive or -negative results, we chose the two target genes, *RdRp* and *N*, to detect viral RNA in clinical samples (Chen et al., 2020; Huang et al., 2020; Pang et al., 2020). To reduce the amplification time, we designed the loop primers. Briefly, six primers targeting eight regions generated a self-priming dumbbell-shaped template upon isothermal incubation with strand-displacing polymerase, resulting in the rapid production of large quantities of the complex amplicon. The specificity of the mRT-LAMP assay was confirmed with genomic RNA from pseudo-viruses of SARS-CoV-2, clinical samples, and other pathogens. The mRT-LAMP detection of the *RdRp* and *N* genes identified SARS-CoV-2 with 100% specificity (**Table 2**).

In previous studies, there have some reports on a molecular diagnostic test for SARS-CoV-2 using RT-LAMP technology. Most of them have used visual inspection of color changes, turbidimetry, and fluorescence dye to analyze RT-LAMP products (Huang et al., 2020; Lu et al., 2020; Park et al., 2020; Yan et al., 2020). However, these techniques have to rely on special instruments and expensive reagents, such as colorimetric indicator, turbidimeter, and fluorescence detector, which may not be readily available in many resource-poor settings. To overcome these drawbacks, a target-specific visual nanoparticle-based lateral flow biosensor (LFB) detection method of easy operation and low-cost (approximately $2 USD) was successfully designed and applied to analyze mRT-LAMP products in the current study. The test result of SARS-CoV-2-mRT-LAMP-LFB provided direct visualization by naked eyes and does not require special instruments. Due to the specificity and elimination of special instruments, the LFB-based LAMP assay could easily apply to various fields (Cheng et al., 2019; Wang et al., 2019). In particular, the LFB applied in this study can simultaneously and visually detect two target genes (*RdRp* and *N*) in a single test.

Compared with RT-PCR method, the mRT-LAMP-LFB technique is more sensitive, time-saving, and cost-saving. The newly developed mRT-LAMP-LFB method was able to detect 20 copies of genomic RNA, which was more sensitive than RT-PCR method (**Figure 5**). The entire detection process, including reaction preparation (approximately 10 min), template preparation (approximately 30 min), isothermal amplification (30 min), and LFB reading (approximately 2 min), could be accomplished within 80 min. The RT-PCR assay, however, requires 2~3 h during the whole process. The running cost of one test, including genomic RNA extraction (approximately $1 USD), LAMP reaction (approximately $3.5 USD), and LFB reading (approximately $2 USD), is estimated to be $6.5 USD, which is getting closer with RT-PCR testing (approximately $7.0 USD). In addition, the advanced technology can decrease labor costs because performing the mRT-LAMP-LFB assay does not require skilled technical personnel. More importantly, the mRT-LAMP-LFB technology has great potential to develop point-of-care (POC) testing in clinical practice. The detection results could be easily judged by the naked eye. The three crimson red bands (CL, TL1, and TL2) appeared indicating positive results, while the negative results only appeared as a crimson red line (CL) in the biosensor. The findings of this study have been applied for a patent from the State Intellectual Property Office of the People’s Republic of China (Patent Application NO. 202010717954. X). The shortcoming of this detection is that the RT-LAMP amplification must be taken out from the reaction tube for LFB detection. There has a risk of contamination with the post-reaction processing of LAMP products. The strict control of the laboratory environment is critical for the reduction of the production of aerosols in experimental processes. Spraying timely 10~15% sodium hypochlorite solution and 70% ethanol after completion of detection is an effective way to overcome nucleic acid contamination in the laboratory. In the current study, the mRT-LAMP-LFB detection results were consistent with the RT-PCR methods in the evaluation of clinical samples. It is indicated that false-positive rates have been effective controlled in our laboratory.

The main limitation of this study is that with the widely spread of SARS-CoV-2 virus, the accuracy of the mRT-LAMP-LFB technology will be affected by the mutations occurring in the primers sequence region of the target genes. So, it is necessary to monitor the mutant sites of the virus genome by whole-genome sequencing. Besides, owning to laboratory biosafety, SARS-CoV and MERS-CoV viruses could not be tested for the specificity of the mRT-LAMP-LFB assay, we used pseudo-virus of SARS-CoV and MERS-CoV as alternatives.

In conclusion, a simple, rapid, and reliable mRT-LAMP-LFB technique based on the *RdRp* and *N* genes was successfully developed for assaying SARS-CoV-2 in the current study. This method could rapidly, reliably, specifically, and sensitively detect SARS-CoV-2. The amplification products were analyzed with LFB, which was objective, rapid, and easily interpretable. Hence, the mRT-LAMP-LFB assay could be considered as a useful method for the reliable and rapid detection of SARS-CoV-2 in clinical samples, especially in resource-constrained regions of the world.

## Data Availability Statement

The original contributions presented in the study are included in the article/**Supplementary Material**. Further inquiries can be directed to the corresponding author.

## Ethics Statement

The study was approved by the Human Ethics Committee of the Second Affiliated Hospital of Guizhou University of Traditional Chinese Medicine (Approval No. TYH2020011) and the Human Ethics Committee of the Zhejiang Hospital (Approval No. 2020 Lin Shen Di (7K) Hao), and complied with the Declaration of Helsinki. All data/isolates were analyzed anonymously.

## Author Contributions

XC, QZ, and SD conceived and designed the study. XC and SD participated in primers design. XC, QZ, BC, YW, and HY contributed to all the laboratory works. BC and HY contributed to the data collection. XC, SL, and QZ performed the statistical analysis. XC wrote the initial draft of the manuscript, and SD revised the manuscript. All authors contributed to the article and approved the submitted version.

## Funding

This work was supported by the Program of Scientific and Technological Project in Guizhou Province (Grant No. [2020]4Y184, [2019]1186 and [2020]4Y197), the Scientific and Technological in Guiyang City (Grant No. Zhu Ke He [2020]-10-5 and [2020]-16-5), the Program of Scientific and Technological Innovation Team of Guizhou Province under Grant (Qian Ke He Platform talent [2018]5606), the Public Welfare Technology Application Research Program of Zhejiang Province (Grant No. LGF21H190001), and the National Natural Science Foundation of China under Grant (81801978).

## Conflict of Interest

The authors declare that the research was conducted in the absence of any commercial or financial relationships that could be construed as a potential conflict of interest.

## Abbreviations

SARS-CoV-2, severe acute respiratory syndrome coronavirus 2; SARS-CoV, severe acute respiratory syndrome coronavirus; MERS-CoV, Middle East respiratory syndrome coronavirus; RT-PCR, real-time reverse transcription-polymerase chain reaction; LAMP, loop-mediated isothermal amplification; LFB, nanoparticle-based lateral flow biosensor; LoD, limit of detection; WHO, World Health Organization; NCBI, National Centre for Biotechnology Information; MG, malachite green; 2^nd^ GZUTCM, Second Affiliated Hospital, Guizhou University of Traditional Chinese Medicine; ZJH, Zhejiang Hospital; GZCDC, Guizhou Provincial Center for Disease Control and Prevention; ZJCDC, Zhejiang Provincial Center for Disease Control and Prevention; 1^st^ ZJUSM, The First Affiliated Hospital, Zhejiang University School of Medicine; Dig, digoxigenin; FAM, 6-carboxy-fluorescein; nt, nucleotide; mer, monomeric unit; TL1, test line 1; TL2, test line 2; CL, control line; NC, negative control; BC, blank control; DW, distilled water; POCT, Point-of-Care testing.
