# Supplementary figures and images for: Rapid and Visual Detection of SARS-CoV-2 Using Multiplex Reverse Transcription Loop-Mediated Isothermal Amplification Linked With Gold Nanoparticle-Based Lateral Flow Biosensor
Source: Front Cell Infect Microbiol. 2021 Jul 14;11:581239. doi: 10.3389/fcimb.2021.581239 (PMC8316814; doi:10.3389/fcimb.2021.581239)

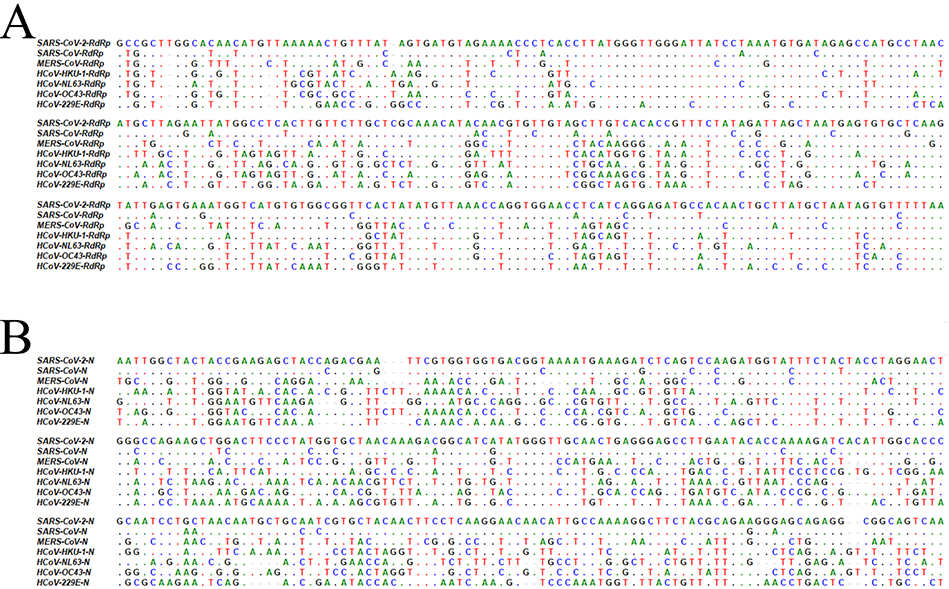

Supplement: Supplementary file 1 [file Image_1.tif]
