# Supplementary material for: Rapid and Visual Detection of SARS-CoV-2 Using Multiplex Reverse Transcription Loop-Mediated Isothermal Amplification Linked With Gold Nanoparticle-Based Lateral Flow Biosensor
Source: Front Cell Infect Microbiol. 2021 Jul 14;11:581239. doi: 10.3389/fcimb.2021.581239 (PMC8316814; doi:10.3389/fcimb.2021.581239)
Supplement: Supplementary file 2 [file Table_1.docx]

**A rapid and visual detection of SARS-CoV-2 using multiplex reverse transcription loop-mediated isothermal amplification linked with gold nanoparticle-based lateral flow biosensor**

Xu Chen^1,2,3Δ^, Qingxue Zhou^4Δ^, Shijun Li^3^, Hao Yan^5^, Bingcheng Chang^1,2^, Yuexia Wang^6^, Shilei Dong^7*^

**Supplementary Materials**

**Results**

*RdRp* and *N* genes sequence alignment among seven human coronaviruses (SARS-CoV-2, SARS-CoV, MERS-CoV, HCoV-HKU-1, HCoV-NL63, HCoV-OC43, HCoV-229E), respectively (**Supplementary Figure 1**).

Comparison of RT-PCR and mRT-LAMP-LFB results for clinical samples and artificial sputum samples (**Supplementary Table 1)**
