# Supplementary material for: Rapid and Visual Detection of SARS-CoV-2 Using Multiplex Reverse Transcription Loop-Mediated Isothermal Amplification Linked With Gold Nanoparticle-Based Lateral Flow Biosensor
Source: Front Cell Infect Microbiol. 2021 Jul 14;11:581239. doi: 10.3389/fcimb.2021.581239 (PMC8316814; doi:10.3389/fcimb.2021.581239)
Supplement: Supplementary file 3 [file Table_2.docx]

**Supplementary TABLE 1: Comparison of RT-PCR and mRT-LAMP-LFB results for clinical samples and artificial sputum samples**

| Sample NO. | RT-PCR result(*Cq* value) | | mRT-LAMP-LFB result | |
| --- | --- | --- | --- | --- |
|  | *RdRp* | *N* | *RdRp* | *N* |
| **Clinical sample** |  |  |  |  |
| Test 1 | 34.26 | 34.80 | + | + |
| Test 2 | 27.19 | 29.53 | + | + |
| Test 3 | 26.45 | 28.58 | + | + |
| Test 4 | 25.54 | 26.73 | + | + |
| Test 5 | 35.09 | 34.48 | + | + |
| Test 6 | 29.09 | 30.44 | + | + |
| Test 7 | 27.03 | 29.14 | + | + |
| Test 8 | 35.20 | 34.50 | + | + |
| Test 9 | 31.31 | 32.50 | + | + |
| Test 10 | 34.94 | 35.39 | + | + |
| Test 11 | 32.71 | 34.46 | + | + |
| Test 12 | 25.11 | 29.50 | + | + |
| Test 13-110 | - | - | - | - |
| **Artificial sputum sample** |  |  |  |  |
| Test 1 | 34.25 | 34.79 | + | + |
| Test 2 | - | - | - | - |
| Test 3 | 31.15 | 33.27 | + | + |
| Test 4 | - | - | - | - |
| Test 5 | - | - | - | - |
| Test 6 | 34.00 | 34.41 | + | + |
| Test 7 | 33.07 | 34.98 | + | + |
| Test 8 | 31.53 | 33.52 | + | + |
| Test 9 | - | - | - | - |
| Test 10 | 33.25 | 35.18 | + | + |
| Test 11 | - | - | - | - |
| Test 12 | 30.63 | 33.61 | + | + |
| Test 13 | 31.64 | 34.20 | + | + |
| Test 14 | 32.52 | 34.67 | + | + |
| Test 15 | 32.89 | 32.43 | + | + |
| Test 16 | - | - | - | - |
| Test 17 | 32.82 | 34.27 | + | + |
| Test 18 | - | - | - | - |
| Test 19 | 32.29 | 34.10 | + | + |
| Test 20 | 32.70 | 34.41 | + | + |
| Test 21 | 32.52 | 33.97 | + | + |
| Test 22 | - | - | - | - |
| Test 23 | 32.70 | 34.46 | + | + |
| Test 24 | - | - | - | - |
| Test 25 | 32.11 | 34.23 | + | + |
| Test 26 | - | - | - | - |
| Test 27 | 33.03 | 34.29 | + | + |
| Test 28 | 32.71 | 34.75 | + | + |
| Test 29 | 32.54 | 34.41 | + | + |
| Test 30 | 32.86 | 33.97 | + | + |
| Test 31 | 32.43 | 34.38 | + | + |
| Test 32 | 32.46 | 34.47 | + | + |
| Test 33 | 32.39 | 33.96 | + | + |
| Test 34 | - | - | - | - |
| Test 35 | - | - | - | - |
| Test 36 | 32.23 | 32.61 | + | + |
| Test 37 | 32.14 | 34.04 | + | + |
| Test 38  Test 39 | - | - | - | - |
| Test 40 | 31.62 | 34.96 | + | + |
| Test 41 | 32.50 | 34.64 | + | + |
| Test 42 | - | - | - | - |
| Test 43 | - | - | - | - |
| Test 44 | 32.95 | 34.43 | + | + |
| Test 45 | - | - | - | - |
| Test 46 | - | - | - | - |
| Test 47 | 33.47 | 34.30 | + | + |
| Test 48 | 32.92 | 34.31 | + | + |
| Test 49 | - | - | - | - |
| Test 50 | 32.83 | 33.89 | + | + |
| Test 51 | - | - | - | - |
| Test 52 | - | - | - | - |
| Test 53 | - | - | - | - |
| Test 54 | - | - | - | - |
| Test 55 | 34.24 | 34.40 | + | + |
| Test 56 | - | - | - | - |
| Test 57 | 33.64 | 34.42 | + | + |
| Test 58 | - | - | - | - |
| Test 59 | 34.38 | 34.71 | + | + |
| Test 60 | 34.32 | 34.11 | + | + |

Cq, quantification cycle; +, Positive; -, Negative
